# Epigenetic regulation of *p62/SQSTM1* overcomes the radioresistance of head and neck cancer cells via autophagy-dependent senescence induction

**DOI:** 10.1038/s41419-021-03539-5

**Published:** 2021-03-05

**Authors:** Myungjin Lee, Hae Yun Nam, Hee-Bum Kang, Won Hyeok Lee, Geun-Hee Lee, Gi-Jun Sung, Myung Woul Han, Kyung-Ja Cho, Eun-Ju Chang, Kyung-Chul Choi, Seong Who Kim, Sang Yoon Kim

**Affiliations:** 1 Department of Otolaryngology, Asan Medical Center, University of Ulsan College of Medicine, Seoul, Republic of Korea; 2 Department of Biochemistry and Molecular Biology, Asan Medical Center, University of Ulsan College of Medicine, Seoul, Republic of Korea; 3 New Drug R&D Center, HLB LifeScience, Hwaseong, Republic of Korea; 4 Biomedical Research Center, Ulsan University Hospital, University of Ulsan College of Medicine, Ulsan, Republic of Korea; 5 Department of Biomedical Sciences, Asan Medical Center, University of Ulsan College of Medicine, Seoul, Republic of Korea; 6 Department of Obstetrics, Gynecology and Reproductive Biology, Michigan State University, Grand Rapids, MI USA; 7 Department of Otorhinolaryngology, Ulsan University Hospital, University of Ulsan College of Medicine, Ulsan, Republic of Korea; 8 Department of Pathology, Asan Medical Center, University of Ulsan College of Medicine, Seoul, Republic of Korea

**Keywords:** Cancer, Cancer therapy

## Abstract

Tumors are composed of subpopulations of cancer cells with functionally distinct features. Intratumoral heterogeneity limits the therapeutic effectiveness of cancer drugs. To address this issue, it is important to understand the regulatory mechanisms driving a subclonal variety within a therapy-resistant tumor. We identified tumor subclones of HN9 head and neck cancer cells showing distinct responses to radiation with different levels of p62 expression. Genetically identical grounds but epigenetic heterogeneity of the *p62* promoter regions revealed that radioresistant HN9-R clones displayed low p62 expression via the creation of repressive chromatin architecture, in which cooperation between DNMT1 (DNA methyltransferases 1) and HDAC1 (histone deacetylases 1) resulted in DNA methylation and repressive H3K9me3 and H3K27me3 marks in the *p62* promoter. Combined inhibition of DNMT1 and HDAC1 by genetic depletion or inhibitors enhanced the suppressive effects on proliferative capacity and in vivo tumorigenesis following irradiation. Importantly, ectopically p62-overexpressed HN9-R clones increased the induction of senescence along with p62-dependent autophagy activation. These results demonstrate the heterogeneous expression of p62 as the key component of clonal variation within a tumor against irradiation. Understanding the epigenetic diversity of p62 heterogeneity among subclones allows for improved identification of the functional state of subclones and provides a novel treatment option to resolve resistance to current therapies.

## Introduction

Cancer arises from the clonal evolution of a single cell during tumor development. However, most human cancers are characterized by extensive intratumoral heterogeneity, containing clonal subpopulations with distinct phenotypes and biological properties. Tumor heterogeneity at diagnosis can be altered by selective pressure from cytotoxic and molecular targeted chemotherapies, which promotes the growth of minor, therapy-resistant tumor cell clones and induces the recurrence of resistance^1–3^. Head and neck cancer is the seventh most common malignancy worldwide^4^, and the majority of patients with cancer receive radiation therapy with curative and adjuvant chemotherapy in early and advanced stage tumors^5^. Despite this, resistance of head and neck cancer to radiotherapy is one of the primary reasons for locoregional recurrence^6,7^. Therefore, it is important to understand the mechanisms associated with the responses of subclones to cancer therapy to improve treatment effectiveness.

Autophagy is a highly conserved nutrient recycling process that removes unnecessary or dysfunctional components via a non-selective bulk incorporation^8^. On the other hand, under metabolic and therapeutic stress conditions, cells drive a selective autophagy that involves adaptors to ensure efficient recognition and sequestration of the cargo within autophagosomes^9,10^, possibly allowing for exploiting autophagy as a therapeutic strategy for targeting cancer cells. We and others have demonstrated that a persistent and high autophagic flux is linked to cellular senescence as a tumor suppressive mechanism^11,12^. p62/SQSTM1 (hereafter p62) is the first identified autophagy adaptor^13,14^. p62 has fundamental functions in tumorigenesis and tumor maintenance owing to its ability to interact with key proteins in various signaling pathways^15–18^. However, the molecular mechanisms controlling p62 expression and the clinical significance of the p62 levels within a tumor are poorly understood.

Functional variation of clones within an individual tumor results, in part, from the presence of genetically different subpopulations^19^. However, epigenetic intratumor heterogeneity plays a relatively more important role in the phenotypic variation of cancer cells with a high degree of genetic homogeneity^1,3^. At the molecular level, epigenetic mechanisms that contribute to tumor heterogeneity include DNA methylation, histone modifications, and chromatin remodeling^20,21^, which events involve several classes of epigenetic regulators that transfer or remove chemical groups to or from DNA or histones, respectively^22,23^.

In this study, we identified two distinct subclones isolated from a primary head and neck tumor. Each clone with distinct radiation sensitivity showed different levels of p62 expression via epigenetic regulation in the *p62* promoter regions. Here, based on the roles in autophagy and senescence induction, we addressed p62 as a candidate therapeutic target to overcome clonal variation to radiation response and to improve prognostic outcomes.

## Materials and methods

### Cell culture

AMC-HN9 cells (Asan Medical Center-Head and Neck cancer 9, referred to as HN9 in this study) were established from an undifferentiated primary head and neck cancer sample isolated from a patient treated at Asan Medical Center^24^ and were authenticated by short tandem repeat sequence analysis. The cells were cultured in Dulbecco’s Modified Eagle’s Medium (DMEM; Invitrogen, Grand Island, NY, USA) supplemented with 10% fetal bovine serum (FBS; Invitrogen), 100 µg/ml penicillin/streptomycin, and 100 µM non-essential amino acids (Invitrogen) at 37 °C in a humidified 5% CO_2_ atmosphere.

### Reagents and radiation

Bafilomycin-A1, 5-Aza-2′-deoxycytidine (5-Aza), and MS-275 (Enzo Life Science, Farmingdale, NY, USA) were purchased from Sigma (St. Louis, MO, USA). For irradiation, a 6 MV photon beam generated by a linear accelerator (CLINAC 600 C; Varian, Palo Alto, CA, USA) at a dose rate of 2 Gy/min was used.

### Single-cell cloning from the tumor cells

HN9 cells were harvested and resuspended in fresh medium to generate a single-cell suspension with a density of ~10 cells/ml. Then, 100 µl of the single-cell suspension was dispensed into each well of a 96-well culture plate. Each well was checked under a phase-contrast microscope, and wells containing only a single cell were marked. When a colony reached confluence, it was transferred to a six-well dish and was maintained until nearly confluent.

### Monitoring of the cell growth rate

Time-dependent cell response profiling was performed using the xCELLigence RTCA DP System (ACEA Biosciences, San Diego, CA, USA) as described by the manufacturer. The growth rate of the cells was measured based on the doubling time obtained using the xCELLigence System.

### Xenograft model

To determine the tumorigenic activity of each clone, flank xenografts were established in 5- to 6-week-old male athymic nude mice (BALB/c *nu*/*nu*) by subcutaneous injection of 1 × 10^7^ cells. When the tumors reached 50–100 mm^3^, mice with established xenografts were stratified by tumor volume and randomized into treatment groups. All groups included five to eight mice, which were euthanized after two weeks of treatment. Animal experiments were approved by the Institutional Animal Care and Use Committee at the Asan Institute for Life Sciences.

### Clonogenic cell survival assay

Cells exposed to different doses of radiation were plated in duplicates at a limiting dilution in six-well plates and incubated in complete medium for 14 days. After staining with crystal violet, clones with >50 cells were counted as positive colonies. The plating efficiency and surviving cell fraction, expressed as a percentage, were calculated relative to those of non-irradiated cells.

### mRFP-GFP/LC3 image analysis

The autophagy reporter plasmid, ptfLC3 (encoding mRFP-GFP-MAP1LC3B; #21074), was purchased from Addgene (Cambridge, MA, USA). Plasmid transfection was performed with Lipofectamine 2000 according to the manufacturer’s instructions. Then, 24 h after transfection, cells were pretreated with bafilomycin-A1 and irradiated, and >20 cells per condition were imaged on a confocal system (Zeiss, LSM 780).

### p62 overexpressing viral infection

The gene encoding p62 was amplified from pcDNA4/TO-HA-p62 (#28027, Addgene) by PCR using Pfu DNA polymerase (ELPIS, Seoul, Korea). The amplified DNA fragment was purified and subcloned into pLenti-suCMV-Rsv-GFP (GenTarget, San Diego, CA, USA) using an EzCloning Kit (Enzynomics, Daejeon, Korea). The lentiviral vector construct was co-transfected with psPAX2 (encoding a packaging plasmid; #12260, Addgene) and pMD2.G (encoding VSV-G envelop plasmid; #12259, Addgene) into HEK293T cells using the Neon Transfection System (Invitrogen). For control and p62 overexpression, supernatants containing the lentivirus were collected and infected into HN9-R clones.

### shRNA lentivirus infection

To generate the lentivirus, pLKO vector or DNMT1-shRNA were transfected together with psPAX2 and pMD2.G into HEK293T cells. For transient infection, cells were infected with the lentivirus in the presence of 5 μg/ml Polybrene (Sigma). The effects of DNMT1-shRNA were measured 48 h after transfection.

### RNA sequencing analysis

The RNA seq analysis was commercially commissioned to Life is Art of Science (Gimpo, Korea). The expression differences of the genes of raw data were evaluated by Skewer ver. 0.2.2^25^, STAR ver. 2.5 software^26^, and Cufflinks software ver. 2.2.1^27^. The *P* values of multiple tests were adjusted using the Benjamini-Hochberg method and the significance level was set at false-discovery rate (FDR) ≤ 0.05 and |log2 FC | ≥ 0.5. Genes that were differentially expressed between clones were analyzed using Fisher’s exact test to determine significant enrichment of Kyoto Encyclopedia of Genes and Genomes (KEGG) pathways.

### Immunoprecipitation

For immunoprecipitation, cells were lysed in RIPA buffer directly followed by sonication. Clarified cell lysates were incubated with the indicated antibodies overnight, and protein A/G beads (Santa Cruz Biotechnology, Inc.) were added for 3–5 h. Beads were washed four times with RIPA buffer. Proteins were eluted in SDS-sample buffer and subjected to western blot analyses. Band intensity was quantified using ImageJ software (National Institutes of Health, Bethesda, MD, USA).

The following antibodies were used: LC3B (L7543; Sigma), p62 (M162-3B; MBL International Corporation, Woburn, MA, USA), DNMT1 (ab13537; Abcam), DNMT3A (ab2850; Abcam), DNMT3B (ab16049; Abcam), HDAC1 (sc7872; Santa Cruz Biotechnology Inc.), HDAC2 (sc7899; Santa Cruz Biotechnology Inc.), HDAC3 (sc376957; Santa Cruz Biotechnology Inc.), NCoR (ab24552; Abcam), acetyled Histone H3 (06-599; Sigma-Aldrich), Histone H3 (ab1791; Abcam), Histone H3K9ac (07-352; Sigma-Aldrich), Histone H3K4me3 (39915; Active Motif), Histone H3K27me3 (ab6002; Abcam), MeCP2 (ab2828; Abcam), and β-actin (A5441; Sigma).

### Chromatin immunoprecipitation (ChIP) assay

A ChIP assay was performed using a Pierce™ Agarose ChIP Kit (Thermo Fisher Scientific, Rockford, USA) according to the manufacturer’s protocols. Precipitated chromatin DNA was recovered and analyzed by PCR. The PCR primer sequences used were P1 forward 5′-GCA CTC ACC TTC CAG GAG GTG-3′, reverse 5′-ATT GTC AAT TCC TCG TCA CTG-3′ or P2 forward 5′-TGT TAT TGA GCT GTA ACT GAA-3′, reverse 5′-CAT GGC CTG TCC ACA CAA CAG-3′.

### Soft-agar assay

Cells (10^5^ cells/well) were mixed with 0.4% agarose in growth medium, plated on top of a solidified layer of 0.5% agarose in growth medium, in a six-well plate, and fed every 3 days with growth medium. After 3–4 weeks, the colonies were dyed with crystal violet (0.01% solution) and counted using OpenCFU software (http://opencfu.sourceforge.net/).

### Statistical analysis

All value were presented as the mean ± standard error. Statistical differences were evaluated using independent-sample two-tailed unpaired Student’s *t*-test or analysis of variance with Bonferroni correction using Microsoft Excel or Prism software. The log-rank test was used for clonogenic survival analysis. The threshold for statistical significance was set at *p* < 0.05. Sample sizes of all experiments were predetermined by calculations derived from our experience. No sample was excluded from the analyses. Investigators were not blinded to the group allocation during the experiment and outcome assessment. Significance values and the number of replicates were indicated in each figure legend.

## Results

### Subclones with different p62 levels within a tumor display distinct responses to irradiation

**Fig. 1:**
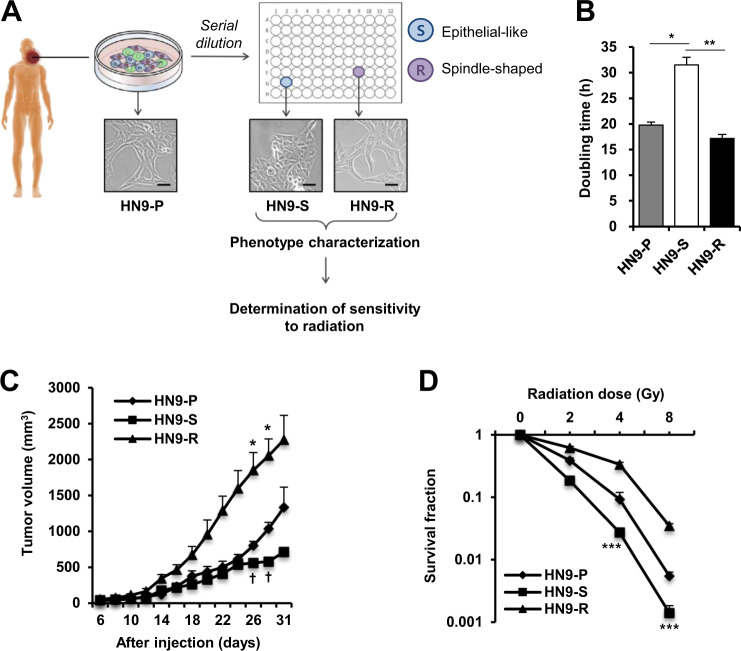
Two subclones derived from a primary head and neck tumor display a distinct growth rate and sensitivity to irradiation. **A** Schematic diagram of isolation and functional characteristic analysis of two subclones (HN9-S and HN9-R) from HN9 parental cancer cells (HN9-P). Scale bars, 20 μm. **B**–**D** The cells were measured on in vitro cell proliferation by doubling time (**B**), growth rate of xenograft tumors (**C**), and clonogenic survival after irradiation (**D**). In Fig. 1C, * and † indicate HN9-R vs. HN9-P and HN9-S *vs* HN9-P with *p* < 0.05, respectively.

We previously showed that prolonged inhibition of mTOR improved sensitivity to radiation of radioresistant cancer cells to be conditioned to enter autophagy-prone senescence^12^. Moreover, we found that not all cells in a given condition exhibited senescence features, suggesting the possibilities of partial responses and the presence of cancer cells that escaped senescence. To define the results, we aimed to characterize subclonal populations of primary head and neck carcinoma, and isolated single clones from the radioresistant HN9 cell line (Fig. 1A). Of 23 single cell-derived clones, we identified two subclones with distinct cell morphologies (HN9-S and HN9-R, Fig. 1A), in which the differences in molecular phenotypes were confirmed. Whereas the spindle-shaped HN9-R clones displayed rapid cell proliferation in culture conditions (doubling time, 17.2 h) and showed an aggressive tumor formation after xenograft (reaching 2000 mm^3^ at 28 days after injection), HN9-S clones with an epithelial-like shape had a lower proliferative ability (doubling time, 31.5 h) and showed a slower tumor growth rate than those of the parental HN9-P cells (Fig. 1B, C). Additionally, HN9-S clones showed lower clonogenic survival rate against irradiation than other clones (Fig. 1D) and induced greater senescence, as evidenced by the percentage of cells positive for senescence-associated β-galactosidase (SA-β-gal) staining (Supplementary Fig. 1A), but little apoptosis was observed in any cells (Supplementary Fig. 1B, C).

**Fig. 2:**
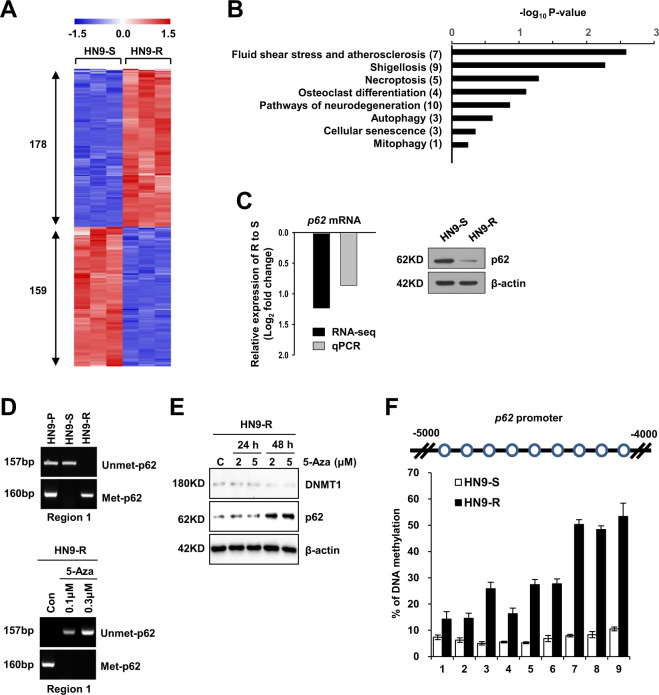
RNA-seq and methylation analyses show that HN9-R is hypermethylated by DNMT1 on the *p62* promoter regions. **A** Heatmap of differentially expressed genes (DEGs) between the HN9-S and HN9-R clones by RNA sequencing analysis (*n* = 3). The number of genes with increased and reduced in HN9-R relative to HN9-S clones were displayed. **B** Enriched Kyoto Encyclopedia of Genes and Genomes (KEGG) pathways associated with *p62* among DEGs between HN9-S and HN9-R clones. The y-axis displays the names and total number of genes of each pathway. DEGs significance was set at FDR ≤ 0.05 and |log2 FC | ≥ 0.5, and pathway significance was set at *p* ≤ 0.01 (Fisher’s exact test). **C** Log_2_ fold change comparison of RNA-seq and qRT-PCR (left) for *p62* and western blotting (right) were used to validate the RNA sequencing data. Negative values indicate that *p62* gene expression was downregulated in HN9-R compared to HN9-S clones. **D** Methylation analysis of the *p62* promoter by methylation-specific PCR in three HN9 cells (upper) and in HN9-R treated with DNMT1 inhibitor, 5-Aza (bottom). **E** Immunoblot analysis of p62 level in HN9-R clones after treatment with 5-Aza. **F** Bisulfite sequencing analysis of the *p62* promoter in HN9-S and HN9-R clones. The *p62* promoter (from forward 4000 to 5000 bp) was analyzed at nine CpG methylation sites using pyrosequencing.

To understand the phenotypic characterization of the two subclones, we performed genome-wide analysis using next-generation sequencing. Two isolated clones were identical to the parental HN9-P cells without any differences in mutations between the clones (99.997% concordance rate, data not shown). However, a transcriptome analysis by mRNA sequencing showed a total of 337 differentially expressed genes (DEGs) between HN9-R and HN9-S clones, and there were 178 and 159 genes with significantly increased or reduced transcripts in HN9-R relative to HN9-S clones, respectively (false-discovery rate (FDR) ≤ 0.05, Fig. 2A and Supplementary Table 1). Of these DEGs, 8 Kyoto Encyclopedia of Genes and Genomes (KEGG) pathways were associated with *p62* (*p* ≤ 0.01, Fig. 2B) and included in pathways with DEGs downregulated at HN9-R clones. In particularly, the level of *p62* gene expression was significantly downregulated by 2.4-fold in resistant HN9-R clones, which difference was verified using qRT-PCR and western blot analysis (Fig. 2C).

### CpG islands of the *p62* promoter are hypermethylated by DNMT1 in HN9-R cells

Gene expression is reversibly altered by epigenetic regulation in clonal evolution, which plays an important role in driving phenotypic variety and tumorigenesis^28,29^. Given the heterogeneous expression of p62 in HN9 cells, CpG islands in the *p62* promoter region were unmethylated with no significant differences between HN9-P and HN9-S clones; however, in HN9-R clones, the *p62* promoter regions were moderately methylated (Fig. 2D). Upon treatment of the DNMT inhibitor 5-Aza-2′-deoxycytidine (5-Aza), the expression of p62 in HN9-R was restored by the hypomethylation of promoter CpG islands (Fig. 2E). Bisulfite sequencing analysis showed that HN9-R clones had substantially higher methylation of nine CpG sites at the *p62* promoter than HN9-S clones (Fig. 2F and Supplementary Fig. 2), indicating that the two clones possessed different patterns of DNA methylation at the *p62* promoter.

**Fig. 3:**
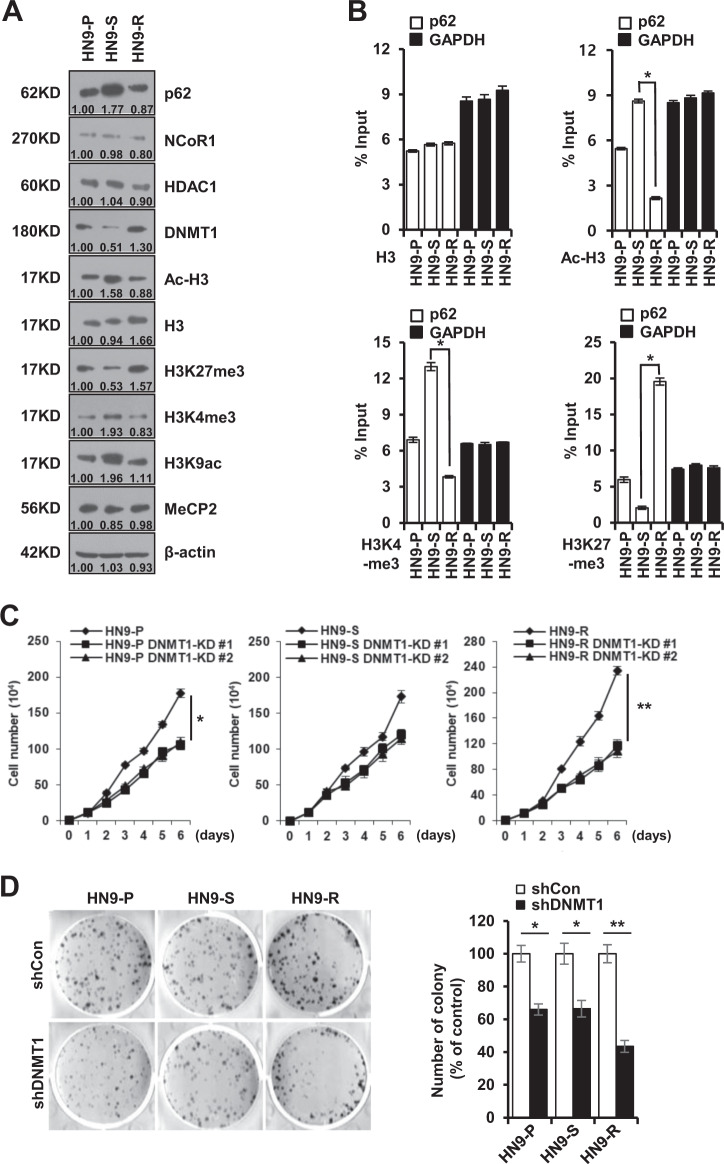
Transcriptional repression of *p62* via DNA methylation increases in vitro tumorigenicity of HN9-R clones. **A** Western blot analysis of p62, NCoR1, HDAC1, DNMT1, and histone modification proteins in HN9 cells. The numbers below the gel lines represent the protein level relative to HN9-P cells, which was determined form the band intensity and normalized by β-actin. **B** Variation in H3 modifications on the *p62* promoter in HN9 cells. The ChIP assay shows transcriptionally active histone markers (Ac-H3 and H3K4me3) enriched on the promoter in HN9-S and an inactive marker (H3K27me3) detected on the promoter in HN9-R. The results obtained from three independent biological replicates were represented as percentage of input (% input). **C**, **D** Proliferative capacity (**C**) and colony formation (**D**) of DNMT1-depleted HN9 cells. Results are presented as means ± standard error of the mean (SEM). *p < 0.05; **p < 0.01.

Next, we compared the levels of co-repressors (NcoR1 and HDAC1) and histone H3 modifications among the HN9 cells to understand the overall expression levels of the *p62* gene by epigenetic regulation^30^. The NcoR1 and HDAC1 levels did not significantly differ among all cell types, but the Ac-H3 and H3K9ac levels specifically increased, with H3K4me3 in HN9-S clones showing high p62 expression (Fig. 3A). The ChIP assay revealed that the levels of transcriptionally active histone markers, Ac-H3 and H3K4me3, were elevated in the *p62* promoter region of HN9-S clones, whereas that of the inactive histone marker H3K27me3 was low (Fig. 3B). Notably, DNMT1 depletion dramatically decreased the cell proliferation and colony formation of HN9-R clones compared to those of HN9-P and HN9-S clones (Fig. 3C, D), implying a therapeutic efficiency of epigenetic regulation by DNMT1 in radioresistant HN9-R clones.

### Epigenetic regulation of p62 increases the sensitivity to radiation of HN9 cells

**Fig. 4:**
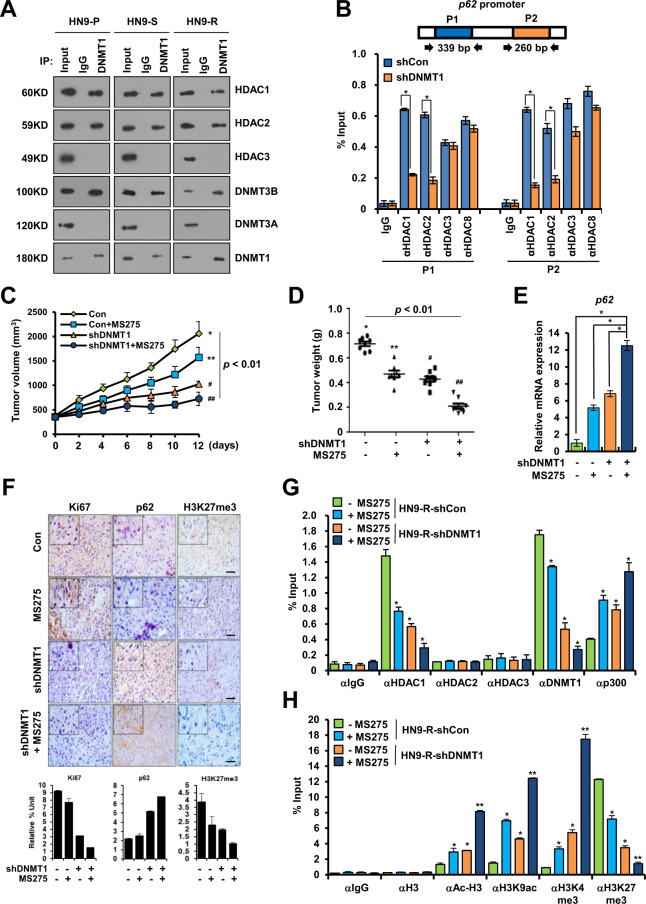
Recovery of p62 expression via the inhibition of DNMT1 and HDAC1 decreases the tumorigenicity of HN9-R clones. **A** Immunoprecipitation analysis of interactions between DNMT1 and HDACs in HN9-P, HN9-S and HN9-R cells. **B** Analysis of the ChIP assay of the *p62* promoter regions (P1 and P2) with antibodies against HDACs in HN9-R clones. The results obtained from three independent biological replicates were represented as percentage of input (% input). **C**, **D** In vivo tumorigenesis (**C**) and tumor weight (**D**) of DNMT1-depleted HN9-R clones with HDAC inhibitor (MS275) treatment. Statistical analyses were determined by ANOVA with Bonferroni correction. Means with different superscript letters are significantly different (*p* < 0.01). **E** *p62* mRNA expression in DNMT1-depleted HN9-R in response to treatment with MS275. **F** Immunohistochemistry and the quantitative analysis of Ki67, p62, and H3K27me3 expression in DNMT1-depleted HN9-R in response to treatment with or without MS275. Scale bars, 50 μm. **G**, **H** ChIP analysis of the *p62* promoter performed with antibodies against HDACs, DNMT1, p300, and H3 histone modification in DNMT1-depleted HN9-R xenograft tumor tissues treated with MS275. Values are means ± standard deviation (SD) for eight mice (**C**–**H**). *p < 0.05; **p < 0.01.

Epigenetic modulation can cure a refractory tumor by inducing the re-expression of essential tumor suppressor genes^31^. DNMT1 represses genes by recruiting the methyl-CpG-binding protein (MeCP2), which gradually recruits HDACs^32^. Co-immunoprecipitation experiment verified that DNMT1 was bound to HDAC1 and HDAC2 in three HN9 cells (Fig. 4A). DNMT1 silencing decreased the recruitment of HDAC1 or HDAC2 to the *p62* promoter of HN9-R clones (Fig. 4B). Also, inhibition of HDAC1 combined with DNMT1 depletion enhanced the inhibitory effect on tumorigenesis (Fig. 4C, D), which corresponded with the level of *p62* mRNA expression (Fig. 4E).

Histological analysis showed that the levels of Ki67 and H3K27me3 expression decreased in DNMT1-depleted HN9-R tumors treated with MS275 and that heterogeneity of p62 substantially decreased in the tumors (Fig. 4F). An in vivo ChIP assay demonstrated that a depletion of DNMT1 efficiently reduced the formation of DNMT1–HDAC1 complexes and subsequently suppressed the recruitment of HDAC1 to the *p62* promoter region, whereas p300 was recruited (Fig. 4G). And MS275 treatment induced the recruitment of Ac-H3, H3K9ac, and H3K4me3 to the *p62* promoter region in DNMT1-depleted HN9-R tumors, while H3K27me3 recruitment decreased as expected (Fig. 4H).

**Fig. 5:**
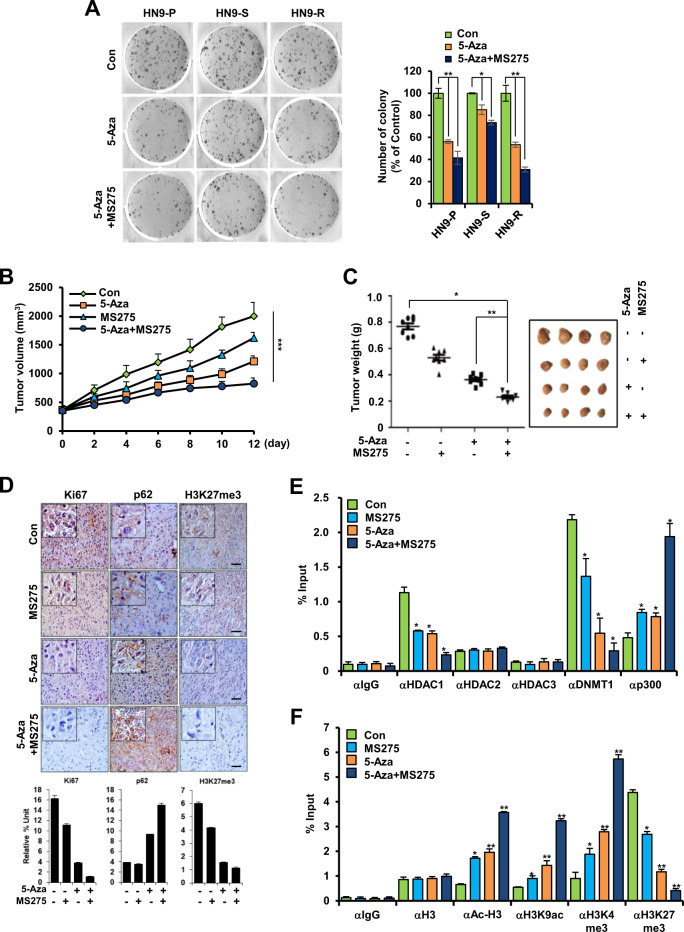
Combined inhibition of DNA methylation and histone deacetylation decreases proliferative capacity and tumorigenesis of HN9-R clones. **A** Colony formation analysis of 5-Aza-treated HN9 cells with or without MS275. **B**, **C** In vivo tumorigenesis of HN9-R clones (**B**) and tumor weight and size of HN9-R xenograft tumors (**C**) after treatment with 5-Aza and/or MS275. **D** Immunohistochemistry and the quantitative analysis of Ki67, p62, and H3K27me3 expression in response to treatment with 5-Aza and/or MS275. Scale bars, 50 μm. **E**, **F** ChIP analysis of the *p62* promoter performed with antibodies against HDACs, DNMT1, p300, and H3 histone modification in HN9-R xenograft tumor tissues treated with 5-Aza and/or MS275. Values are means ± standard deviation (SD) for eight mice (**B**–**F**). *p < 0.05; **p < 0.01; ***p < 0.005.

Likewise, co-treatment of 5-Aza and MS275 impaired colony formation compared to that in the cells treated only with 5-Aza (Fig. 5A) and significantly suppressed tumor volume and weight compared to those of the non-treated control (Fig. 5B, C). Histological analysis showed that the levels of Ki67 and H3K27me3 expression decreased in 5-Aza- and MS275-treated HN9-R tumors and that p62 expression dramatically increased (Fig. 5D). Additionally, co-treatment significantly dissociated the DNMT1–HDAC1 complexes (Fig. 5E) and induced an enhanced recruitment of Ac-H3, H3K9ac, and H3K4me3 and a decrease of H3K27me3 to the *p62* promoter region (Fig. 5F). Corresponding with these findings, the implanted tumor volume and weight significantly decreased in the co-treatment group of 5-Aza/MS275 and radiation compared to the radiation-only group (Supplementary Fig. 3A, B), which was correlated with an increased enrichment of H3K9ac and H3K4me3 and a decline of H3K27me3 to the *p62* promoter region (Supplementary Fig. 3C–E).

### p62 overexpression improves the sensitivity to radiation of resistant cancer cells

To confirm the mechanism by which the level of p62 determines the radioresistance of HN9 cancer cells, we observed whether the maintenance of p62 level mediates efficient transition to the senescent state through autophagy, which can improve responsiveness to irradiation in HN9-R clones, consistent with previous findings^11,15^.

**Fig. 6:**
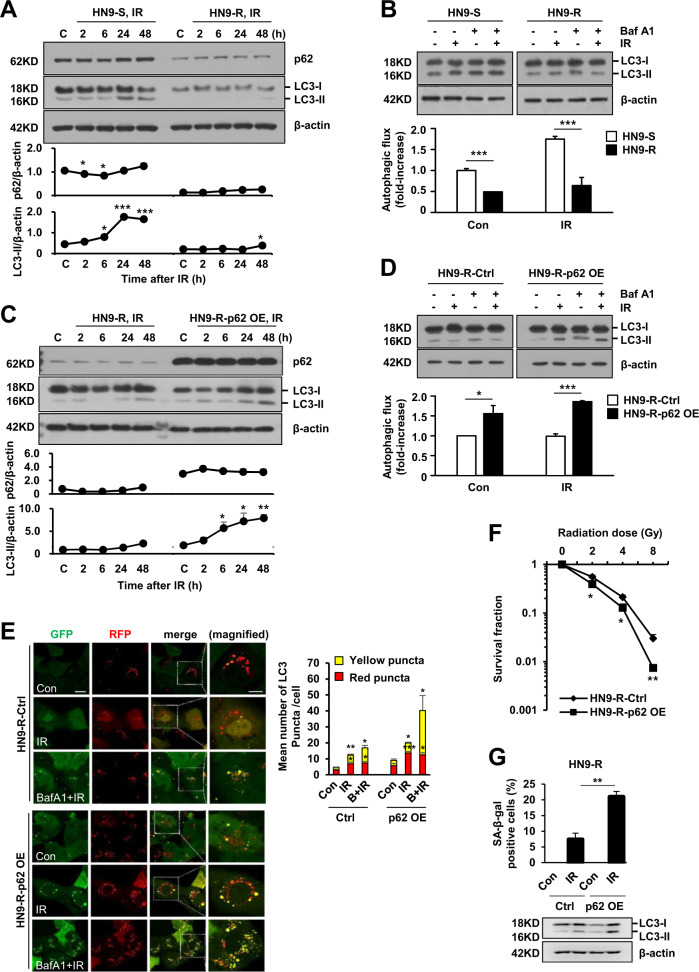
Overexpression of p62 increases autophagic flux and senescence to responses to radiation in HN9-R clones. **A**–**D** Turnover of LC3 over time after irradiation (IR) at 4 Gy (**A**, **C**) and accumulated amounts of LC3-II at 24 h after 4 Gy of IR in the presence of bafilomycin-A1 (Baf A1, 1 nM, 4 h; **B**, **D**) in HN9-S *vs* HN9-R clones (**A**, **B**) or vector control *vs* p62-overexpressed HN9-R clones (**C**, **D**) analyzed by immunoblotting. In **B** and **D**, the relative ratio of LC3-II to β-actin was used as an indicator of autophagic flux, which was calculated by comparing the cells treated with IR and Baf A1 to that with IR only. Results are presented as means ± standard error of the mean (SEM). Western blots are representative of three independent experiments. **E** Measurement of autophagic flux by mRFP-EGFP/MAP1LC3B puncta assay to irradiation (4 Gy, 6 h) after pretreatment of Baf A1 (10 nM, 1 h) in vector control and p62-overexpressed HN9-R clones. Graph represents quantification of RFP-positive, GFP-positive autophagosomes (yellow puncta) and RFP-positive, GFP-negative autolysosomes (red puncta). Scale bars, 20 μm in GFP image and 10 μm in magnified image. **F**, **G** Clonogenic survival (**F**) and percentage of cells positive for senescence-associated beta-galactosidase (SA-β-gal) staining of p62-overexpressed HN9-R after irradiation of 4 Gy (**G**). All results were obtained from at least three independent experiments. Results are presented as means ± standard error of the mean (SEM). *p < 0.05; **p < 0.01; ***p < 0.005.

**Fig. 7:**
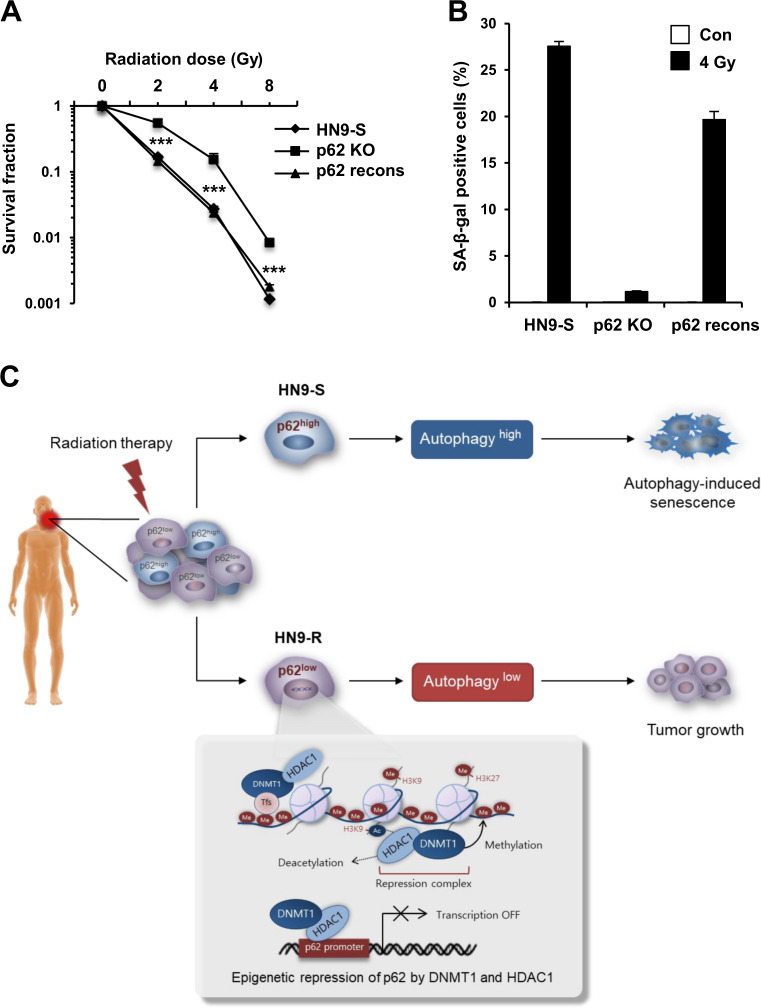
Knock-out of p62 expression decreases a sensitivity to radiation and senescence induction in HN9-S clones. **A**, **B** HN9-S cells were totally ablated of endogenous p62 using the CRISPR/Cas9 system (p62 KO), after which a reversal construct of wild type p62 was transiently transfected to p62 KO cells (p62 recons). Clonogenic survival (**A**) and percentage of cells positive for SA-β-gal staining (**B**) in HN9-S and p62 gene-edited HN9-S cells after irradiation (*n* = 3). Results are presented as means ± standard error of the mean (SEM). ***p < 0.005. **C** Overview of the different epigenetic regulation of *p62* expression and the survival responses against irradiation in subclones, HN9-R and HN9-S, within a radio-resistant head and neck cancer tumor.

When irradiated, HN9-S showed a prominent accumulation of LC3-II after 24 h, whereas HN9-R showed a slight increase in the level of LC3 (Fig. 6A). On the other hand, the level of p62 was not dramatically changed following irradiation both in HN9-S and R clones, although transiently reduced during the early hours (2–6 h) in HN9-S clones (Fig. 6A), which made us to take turnover of LC3 to LC3-II as an autophagic flux reporter rather than p62 level at least in HN9 cells. Furthermore, in the presence of bafilomycin-A1 that functionally inhibits autolysosomal activity, HN9-R clones had less accumulated LC3-II than HN9-S clones and the cells did not show a marked increase of autophagic flux after irradiation (Fig. 6B). However, the overexpression of p62 in the HN9-R clones increased an accumulation of LC3 over time after irradiation (Fig. 6C), and induced a massive accumulation of LC3-II in the presence of bafilomycin-A1 compared to that in the control (Fig. 6D). Also, the p62 overexpressing cells exposed to irradiation increased the number of RFP-positive, GFP-positive puncta (Fig. 6E) that visually proved the effective transition from autophagosomes to autolysosomes^33^. In particular, when considering the inborn radiation resistance of HN9-R clones and the fractionated application of radiation therapy in clinic, clonogenic survival and SA-β-gal staining data indicated that p62 overexpression can significantly improve the response to radiation in the cells (Fig. 6F, G). Furthermore, we could consolidate our findings by the evidences that the ablation of p62 in HN9-S clones lowered autophagic flux and caused an resistance against irradiation and a reduction of senescence, which were restored by exogenous overexpression of p62 (Fig. 7A, B and Supplementary Fig. 4A, B).

Taken together, the two clones isolated from a primary tumor showed distinct differences in the responses to radiation, and this difference was associated with p62-dependent autophagic flux and senescence. Additionally, p62 was maintained in a transcriptionally inactive state by the enrichment of HDAC1–DNMT1 complexes in the *p62* promoter region in HN9-R clones; thus, simultaneous inhibition of DNMT1 and HDAC1 enhanced the responses to irradiation by decreasing epigenetic repression of the *p62* expression (Fig. 7C).

## Discussion

Intratumoral heterogeneity has a major effect on therapeutic outcomes because subpopulations consisting of cancer cells require selective and specific^34^. However, major therapeutic strategies have not considered the functional diversity of clones or the frequency of actionable alterations. This study demonstrates that, given the heterogeneous expression of p62 in subclones within a tumor, combined inhibition of epigenetic modifiers (DNMT1 and HDAC1) reversed the histone pattern and increased p62 expression, which significantly improved the sensitivity to radiation of HN9-R clones.

To date, many studies have shown that upregulation of p62 has fundamental functions in tumorigenesis^17,35,36^. p62 accumulation effectively induces carcinogenesis in autophagy-competent livers by protecting precancerous cells from oxidative stress-induced death^17^. Additionally, the loss of p62 in the stroma regulates cellular redox via an mTORC1/c-Myc pathway, which generates an environment conducive to inflammation and in turn maintains a pro-tumorigenic metabolic state^35^. Unlike the roles of p62 in tumor initiation and progression, our findings imply the clinical significance of p62 targeting in terms of heterogeneous tumor treatment, providing an integrative understanding of p62 as a major determinant of the reactivity to cancer therapy.

In particular, to monitor autophagic flux appropriately, we observed a conversion of LC3-I to LC3-II over time, an amount of accumulated LC3-II in the presence of bafilomycin A1 and immunofluorescence images of RFP-GFP-LC3 reporter, but not p62 degradation. Although the p62 is widely used as an indicator of autophagic degradation, it was reported its inconsistency of autophagic activity. The level of p62 protein is determined by its transcriptional upregulation in some cases and dynamically regulated depending on cell contexts^37,38^. Furthermore, given that the transcriptional level of p62 is epigenetically regulated differently in each clone, we choose to adopt LC3B as a more reliable indicator for autophagy flux in our experiments with other complementary methods.

Prior to isolating HN9 cells, the patient had never been exposed to any cancer treatment and showed an extremely poor prognosis after tumor resection, which indicates that clonal variety within a tumor may be acquired during tumor development rather than induced by the selective pressure of anti-cancer agents. The gene-based analyses revealed that functional variation of clones was controlled by the distinct epigenetic mechanism. In addition, each clone expanded into a heterogeneous culture that fully recapitulated the parent cell-type heterogeneity in in vivo transplants (Supplementary Fig. 5), as implicated in the previous study^39^.

High tumor heterogeneity has been associated with shorter progression-free survival^40^ and is a potential prognostic feature in a variety of malignancies^41,42^. One potential approach to overcome intratumoral heterogeneity is to target multiple pathways simultaneously. In our experimental design, the targeting of both p62-mediated autophagy and a conversion of senescence to apoptosis could act to limit the selection of resistance mechanisms.

In conclusion, our findings indicate that clonal variation of p62 expression induces inconsistent responses to irradiation, and concurrent treatment with epigenetic drugs suppresses the recurrence of resistant tumor cells and reverses the resistance. Furthermore, defining the relationship between epigenetic heterogeneity of *p62* expression and tumor progression can lead to new diagnostic tools and treatment strategies to improve the therapeutic outcomes further.

## Supplementary information

Supplementary Fig 1

Supplementary Fig 2

Supplementary Fig 3

Supplementary Fig 4

Supplementary Fig 5

Supplementary Figure Legends

RNA sequencing DEGs list
