# Supplementary figures and images for: Epigenetic regulation of p62/SQSTM1 overcomes the radioresistance of head and neck cancer cells via autophagy-dependent senescence induction
Source: Cell Death Dis. 2021 Mar 5;12(3):250. doi: 10.1038/s41419-021-03539-5 (PMC7935951; doi:10.1038/s41419-021-03539-5)

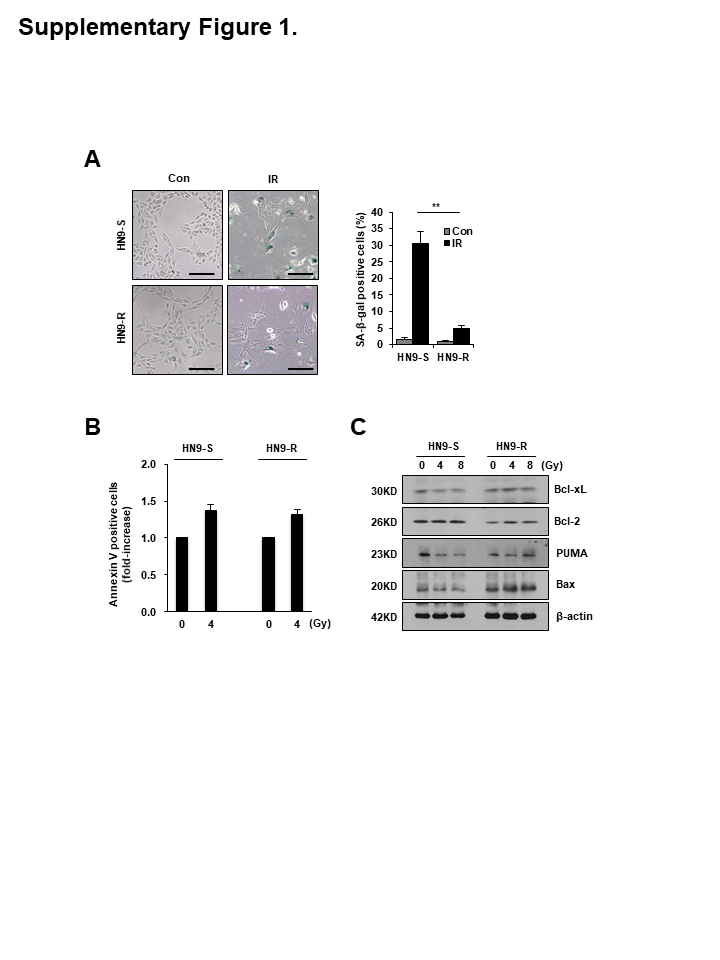

Supplement: Supplementary file 1 — Supplementary Fig 1 [file 41419_2021_3539_MOESM1_ESM.tif]

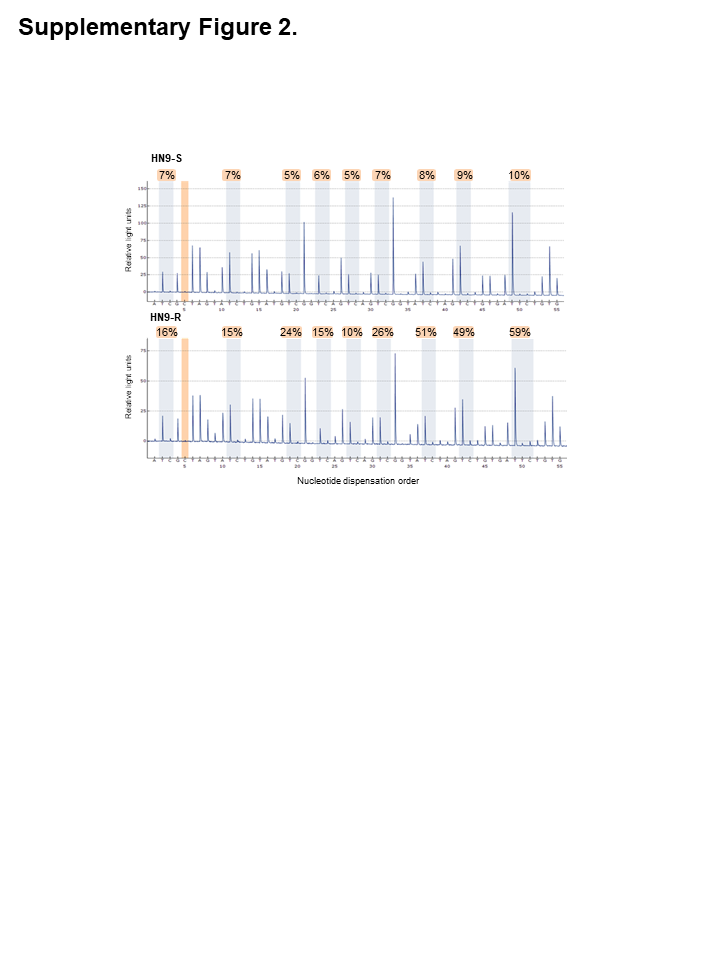

Supplement: Supplementary file 2 — Supplementary Fig 2 [file 41419_2021_3539_MOESM2_ESM.tif]

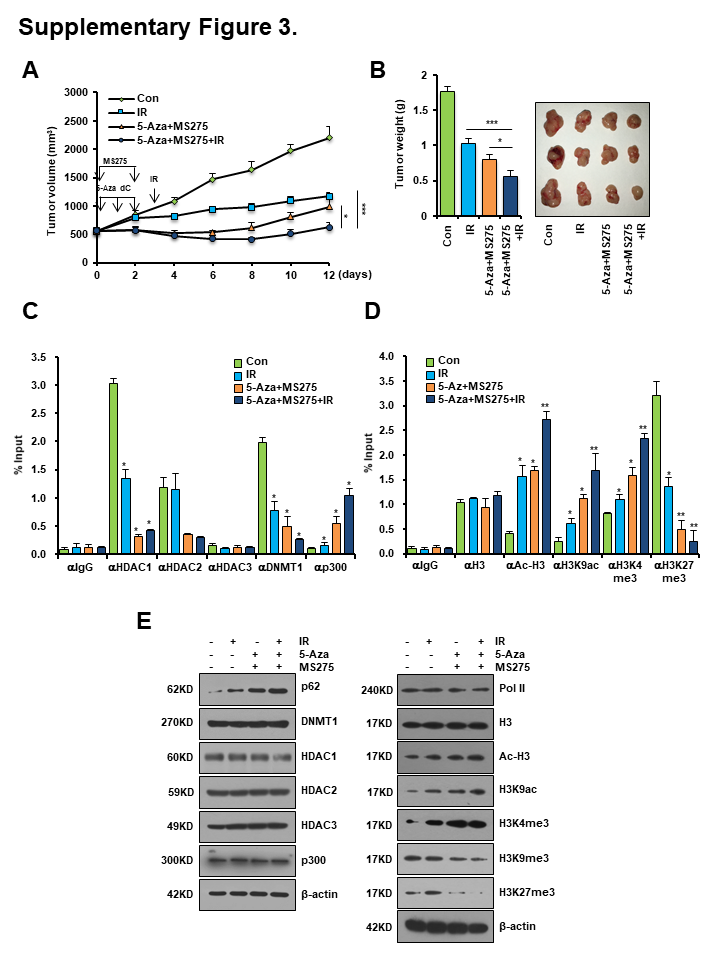

Supplement: Supplementary file 3 — Supplementary Fig 3 [file 41419_2021_3539_MOESM3_ESM.tif]

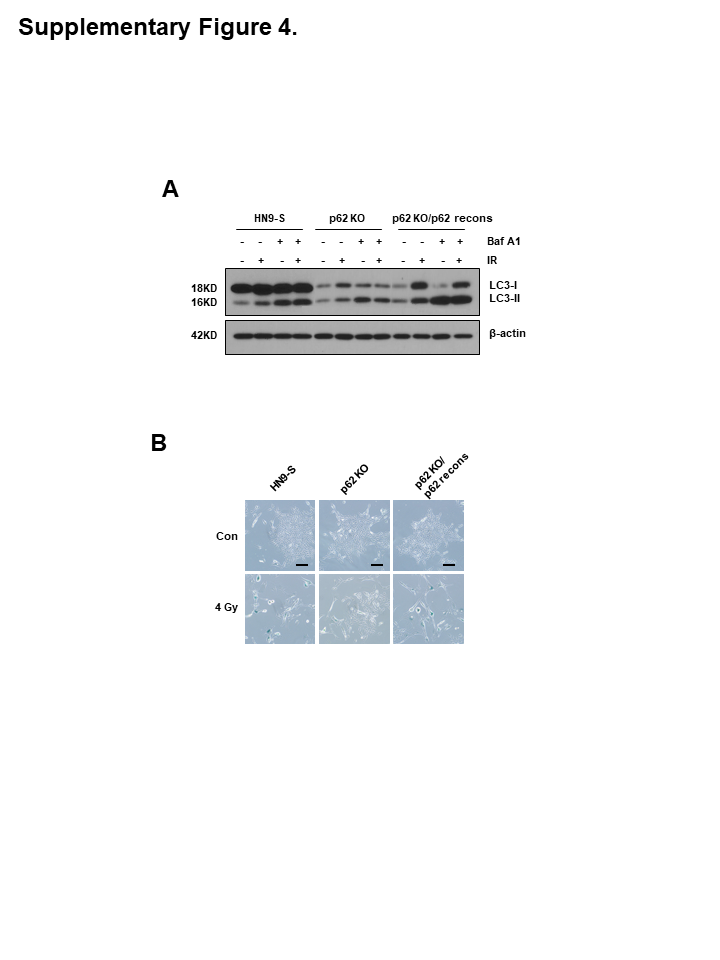

Supplement: Supplementary file 4 — Supplementary Fig 4 [file 41419_2021_3539_MOESM4_ESM.tif]

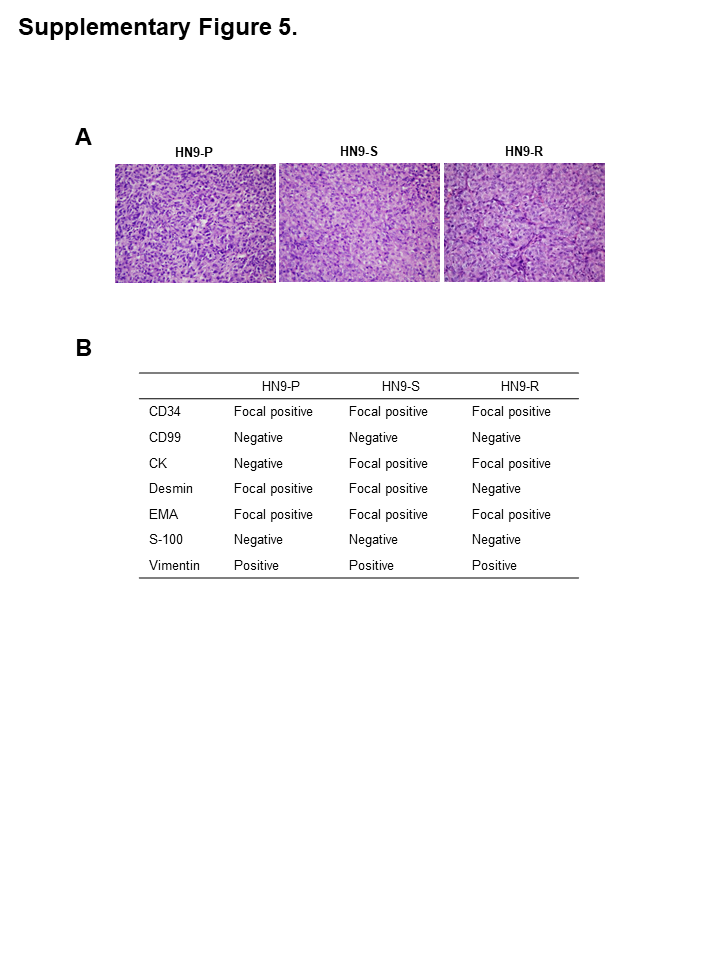

Supplement: Supplementary file 5 — Supplementary Fig 5 [file 41419_2021_3539_MOESM5_ESM.tif]
