# Supplementary material for: Epigenetic regulation of p62/SQSTM1 overcomes the radioresistance of head and neck cancer cells via autophagy-dependent senescence induction
Source: Cell Death Dis. 2021 Mar 5;12(3):250. doi: 10.1038/s41419-021-03539-5 (PMC7935951; doi:10.1038/s41419-021-03539-5)
Supplement: Supplementary file 6 — Supplementary Figure Legends [file 41419_2021_3539_MOESM6_ESM.docx]

**Supplementary Figure Legends**

**Supplementary Fig. 1 Two clones isolated from a primary head and neck tumor differ in senescence induction after irradiation, but hardly induce apoptosis.** **A** Percentage of cells positive for SA-β-gal staining after irradiation at 4 Gy in HN9-S and HN9-R clones (n = 3). Scale bars, 100 µm. **B, C** To evaluate apoptosis after irradiation, the cells were incubated with annexin V at 72 h after irradiation and analyzed using flow cytometry. The percentage of annexin V-positive cells was represented as fold-increases relative to non-irradiated cells (**B**). The cells exposed to irradiation were subjected to western blotting for indicated proteins (**C**).

**Supplementary Fig. 2 DNA methylation analysis of *p62* promoter by pyrosequencing in HN9-S and HN9-R clones.** Quantitative analysis of methylation of nine CpG sites at the *p62* promoter (from forward 4000 to 5000 bp) by pyrosequencing. In pyrosequencing reaction, a ‘C’ is incorporated when the template CpG is methylated, while a ‘T’ is incorporated if the template CpG is unmethylated. In the resulting pyrogram, the ratio of C:T reflects the portion of methylation at the specific region of CpG sites assessed. Ref sequence: **C**GCCAAGAC**C**GGCCAG**C**GGGG**C**GG**C**G**C**GGGGGGAT**C**GGCC**C**GC ACCCTC**C**GCCTGC; Sequence to analyze: GTTAAGAT**Y**GGTTAG**Y**GGGG**Y**GG**Y** G**Y**GGGGGGAT**Y**GGTT**Y**GTATTTTT**Y**GTTTGT.

**Supplementary Fig. 3 Combined inhibition of DNMT1 and HDAC1 enhances a sensitivity to radiation of HN9-R clones. A, B** *In vivo* tumorigenesis (**A**) and tumor weight and size of HN9-R xenograft tumor (**B**) after treatment with 5-Aza, MS275, and irradiation (IR). **C, D** ChIP analysis of the *p62* promoter performed with antibodies against HDACs, DNMT1, p300, and H3 histone modification in HN9-R tumor tissues treated with 5-Aza, MS275, and IR. The results were represented as percentage of input (% input). **E** Western blot analysis performed with antibodies against p62, DNMT1, HDACs, p300, and histone modification in HN9-R xenograft tumor treated with 5-Aza, MS275, and IR. Results of immunoblot analyses are representative of two independent experiments. **p* < 0.05; ***p* < 0.01; ****p* < 0.005.

**Supplementary Fig. 4 The level of p62 expression regulates autophagic flux and induction of senescence to radiation in HN9-S clones.** **A, B** HN9-S cells were totally ablated of endogenous p62 using the CRISPR/Cas9 system (p62 KO), after which a reversal construct of wild type p62 was transiently transfected to p62 KO cells (p62 KO/p62 recons). The cells were exposed to irradiation (4 Gy) after pretreatment of Baf A1 (1 nM, 4 h) and after 24 h were analyzed by western blotting to measure autophagic flux (**A**). Results of western blotting are representative of three experiments. The cells exposed to irradiation were stained with SA-β-gal to observe senescence induction (n = 3, **B**). Scale bars, 50 µm.

**Supplementary Fig. 5. Histopathology and immunohistochemical analysis of xenograft tumor tissues of HN9-P, HN9-S, and HN9-R cells. A** Xenograft tumor tissues of three HN9 cells were stained with hematoxylin and eosin (H&E) stain (×400). **B** Immunohistochemical analysis was performed with the indicated markers to compare the pathological differentiation of each tumor. CK, cytokeratin; EMA, epithelial membrane antigen.
